# Fluorescent impurity emitter in toluene and its photon emission properties

**DOI:** 10.1038/s41598-018-26686-w

**Published:** 2018-05-29

**Authors:** Cong Tai Trinh, Jiwon Lee, Kwang-Geol Lee

**Affiliations:** Department of Physics, Hanyang University, Seoul, 04763 Republic of Korea

## Abstract

Single fluorescent emitters like colloidal quantum dots or single molecules are usually prepared in solutions and spin-coated onto cover glasses for studying. Toluene has been a widely used solvent in such studies. Here, we report on a fluorescent impurity emitter contained in toluene and its optical properties. The emission spectra of the single emitters show multiple peaks with the main peak varying from 2.03 eV (610 nm) to 2.14 eV (580 nm) and a red-shifted side peak with an average separation of 167 meV from the main peak. The emitted photons show a strong anti-bunching with a fluorescence lifetime of a few nanoseconds. They show very fast blinking behavior which cannot be properly detected by time-trajectory of photoluminescence intensity. An analysis based on the second-order correlation functions reveals that a three-level model can explain our measurements well and that the blinking transition time ranges only a few tens of microseconds. This single emitter in toluene is clearly distinguished from the fluorescent centers in the cover glass by their respective emission spectra. The single emitters in the cover glass also exhibit fast blinking behavior. These background emitters should be carefully identified and distinguished while studying the single fluorescent emitters.

## Introduction

Single fluorescent emitters play a central role in numerous current scientific studies and applications. Single emitters, for instance, can serve as fluorescent markers in bio-imaging^1^. In quantum optical applications, single emitters are proposed as single-photon sources which are in huge demand in a range of proposed schemes of quantum information, computing, and metrology^2,3^. Owing to an enormous diversity of potential applications, the photo-physical properties of single emitters such as single molecules^4–7^ or colloidal quantum dots (QD) have been extensively studied over several decades^8–13^. With the development of photon detection technologies and analyses schemes, it is nowadays common to look into the optical properties of single emitters at a very low signal level. Under this situation, the weak backgrounds should be carefully distinguished from the target signals.

Samples of the single fluorescent emitters have been usually prepared in solutions and then spin-coated on substrate^4,6,7,9,14–16^. In this study, we report on a fluorescent impurity emitter contained in toluene solutions. The fluorescence lifetime of this impurity is a few nanoseconds and the emitted photons show a strong anti-bunching nature. The emission spectra under 532-nm laser excitation clearly show multiple peaks. The main peak position varies from 2.03 eV (610 nm) to 2.14 eV (580 nm) and a red-shifted side peak is observed, which is separated from the main peak by 167 meV on an average. We also perform blinking study of this single fluorescent emitter by two different methods: photoluminescence (PL) intensity trajectory method and second-order correlation function *g*^(2)^(*τ*) method. Our results reveal that this emitter exhibits very fast blinking behavior with an average duration of about 15 μs in the OFF state, which cannot be properly analyzed by the time-trace method. By analyzing the *g*^(2)^ curves, a three-level energy scheme could well explain the experiments. This fluorescent emitter in toluene is distinguished from the fluorescent centers in the cover glass by their PL spectra. With its discovered photon emission properties, there is a high possibility for it to be misinterpreted as the other “target” emitter in many single emitter studies.

## Results

**Figure 1:**
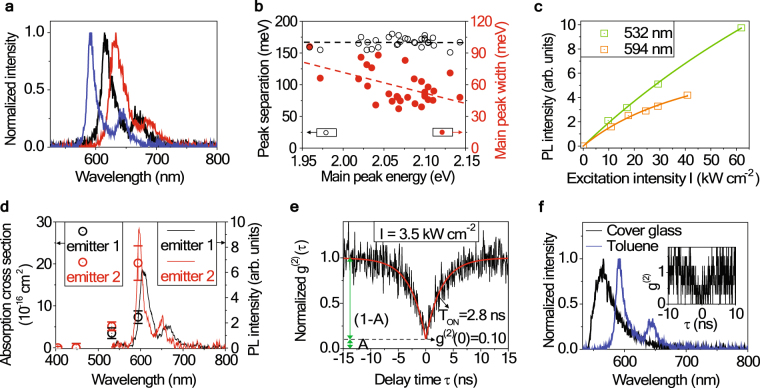
Photoluminescence properties of single emitters from toluene evaporated on cleaned cover glasses. (**a**) The PL spectra of three selected single emitters clearly show two emission peaks. (**b**) The separation between the main and the side peak (black open circles) and the FWHM of the main peak (red solid circles) plotted as a function of the main peak energy. Black dashed line indicates the average value of the peak separation, and the red dashed line is a guide for the eye. (**c**) The PL intensity curves as a function of the pump intensity at excitation wavelengths of 532 nm (green) and 594 nm (orange). Squares are experimental values and solid lines are the fitting. (**d**) Absorption cross section values (open circles with error bars) of two selected emitters. Solid lines are the emission spectra measured at 532 nm excitation. (**e**) Normalized second-order correlation function *g*^(2)^(*τ*) at a short time scale (|τ| < 100 ns). *g*^(2)^(0) = 0.10 with background subtraction. (**f**) Spectral comparison between the impurities in toluene (blue curve) and in cover glass (black curve). The inset is the *g*^(2)^(*τ*) of a defect center in cover glass.

We investigated the photon emission properties of the fluorescence centers on a cover slip where a few drops of toluene were dried. The sample was excited with a continuous wave (cw) 532 nm laser. Details of the sample preparation and the detections are given in the Methods section. Figure 1a shows photoluminescence spectra of three selected emitter centers. They commonly show two peaks – a main peak and a red shifted side peak. The main peak position varies for each particle and ranges from 2.03 eV (610 nm) to 2.14 eV (580 nm) for 30 individual centers. The average separation between the main and the side peaks is 167 meV, as shown by the black dotted line in Fig. 1b. Red solid circles in Fig. 1b show the FWHMs (full width at half maximum) of the main peaks as a function of the main peak energy. Although data points are spread, there is a tendency of broader widths for peaks at lower energies (longer wavelengths). This suggests that the difference in the PL peak positions of individual emitters is probably caused by different local environment (effective refractive index) for each emitter. It was not possible to measure the continuous absorption spectrum of the emitter, but we measured the absorption cross-section at four wavelengths of 405, 448, 532, and 594 nm. Figure 1c shows the PL intensity curves as a function of the pump intensity obtained at 532 nm (green) and 594 nm (orange) excitation wavelengths. Solid lines are the fitting function of $\sim \frac{\sigma j}{{k}_{21}+\sigma j}$, where *k*_21_, *σ*, and *j* are the fluorescence decay rate, the absorption cross section of the emitter, and the input photon flux at the position of the emitter, respectively. Obtained absorption cross section values for two selected emitters are displayed together with the emission spectra in Fig. 1d. We found negligible fluorescence (absorption) at shorter wavelengths of 405 nm and 448 nm. This contradicts the continuous absorption at short wavelengths for semiconductor nano-crystals^17,18^. Though it is not clear with limited number of data points, it seems the absorption spectrum is symmetric to the emission spectrum in the energy domain, and rapidly banishes at shorter wavelengths as a common feature of single molecules^19,20^. The average absorption cross-section values of five emitters at 532 nm and 594 nm are obtained as (4.3 ± 1.6) × 10^−16^ cm^2^ and (12.1 ± 6.0) × 10^−16^ cm^2^, respectively. This also agrees with typical absorption cross section values of single molecules.

Figure 1e displays a normalized *g*^(2)^(*τ*) of a selected center at an excitation intensity of *I* = 3.5 kW/cm^2^. After subtracting the background, including the dark counts of the detector^21^, the measured *g*^(2)^(*τ*) was fitted with a double-side exponential ${g}^{(2)}(\tau )=A+(1-A)\times (1-\exp (\frac{|\tau |}{{T}_{on}}))$, as shown in Fig. 1e. A strong anti-bunching with *g*^(2)^(0) = 0.10 suggests that this single emitter can be a good single photon source.

## Discussion

In order to find whether the above discussed emitters indeed come from toluene instead of being the silica-based impurities of the cover glass, we compared the two emitters through the following measurements. For the cleaned cover glass sample, the emitter centers survive only a few seconds. The emission peak is at a shorter wavelength (~564 nm) and broader in bandwidth compared to that of the emitter from toluene. The emission from the cover glass also showed photon anti-bunching as can be seen in the inset of Fig. 1f. These properties of the emitter in the cover glass are consistent with those cited in a recent report by Rabouw *et al*.^22^. From the noticeable differences in their PL spectra shown in Fig. 1f, we conclude that the two are different kinds of emitters. We observed the PL spectrum shown by the blue curve in Fig. 1f only when we put toluene solution onto cover glass and dried it. Furthermore, our target emitters also appeared when we tried with toluene solutions from different manufacturers (Sigma Aldrich, Daejung). Hence, we concluded that the single emitter is an impurity in toluene and not in the cover glass. It is worth mentioning that the single emitters in this work are very similar to CdZnSe/ZnSe QDs reported by Wang *et al*.^16^. In their studies, they characterized two samples by spin coating a dilute solution of CdZnSe/ZnSe QDs in *toluene* or CdSe/ZnSe QDs in *water* onto a quartz coverslip. The main contrast between the two samples, except the compositions of QDs, was the solvent; *toluene* or *water*. Hence, we believe that they observed the single emitter from the impurities of toluene and not from the CdZnSe/ZnSe QDs^16^ or silica centers as mentioned by Rabouw *et al*.^22^.

**Figure 2:**
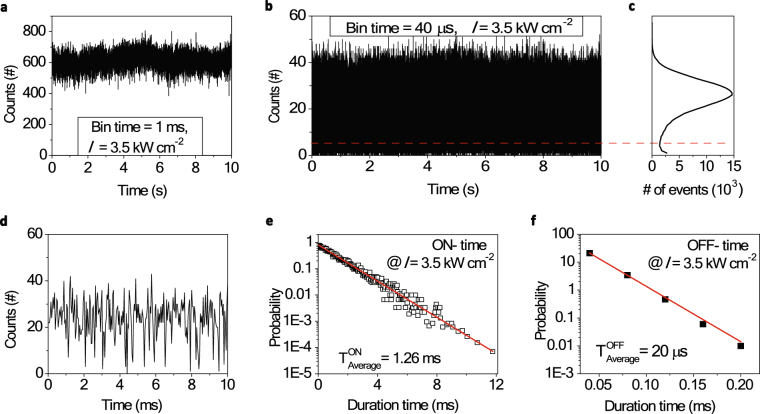
Blinking study by the PL intensity trajectory. (**a**) PL intensity trajectory of a single emitter with a time bin size of 1 ms. (**b**) The same data in (**a**) but with a smaller time bin size of 40 μs. (**c**) Histogram of the photon count events of the time trajectory in (**b**). (**d**) A zoomed in intensity trajectory of (**b**). Probability distributions of (**e**) “ON” and (**f**) “OFF” time durations presented in semi-log scale. Single exponential fittings (red lines) are applied.

As the next step, we characterized our target emitters further on their photoluminescence intermittence, often called blinking. This blinking can restrict the photon emission capability of single emitters severely and, hence, it has been an intense research topic for several decades since its first observation by Cook *et al*.^23^ in the middle of the 1980s. Figure 2a shows the PL intensity trajectory of the same emitter as the one in Fig. 1e. The excitation intensity was set at a value of *I* = 3.5 kW/cm^2^. With a time bin size of 1 ms, which is a typical time bin size used in the blinking studies of QDs^10,24^, the emitter seemed to be non-blinking. However, when we decreased the time bin size down to 40 μs, as shown in Fig. 2b, the intensity trajectory started to show the corresponding “OFF” states. In our measurements, the detection photon counts as high as 6 × 10^5^ cps could be attained; hence, the time bin size could be reduced to few tens of microseconds. The histogram of the photon number per time-bin shown in Fig. 2c clearly shows the “ON” and “OFF” contributions. In the zoomed in trajectory shown in Fig. 2d, there are short “OFF” durations. The average photon number per time-bin of 40 μs is 27 and the Poisson statistics cannot explain the small photon numbers below 11 (outside 3 times of standard deviation) without assuming the “OFF” states, the blinking. Figure 2e and f show the probability distributions of the “ON” and “OFF” durations in semi-log plots^25^ (for details, please refer to the Methods section). Both show clear exponential decays with time constants of 1.26 ms (for ON) and of 20 μs (for OFF), respectively. These time constants indicate the average durations of the “ON” and “OFF” states. The average OFF time duration of 20 μs found in Fig. 2f is below the minimum time bin size of 40 μs applied in our analysis; thus, the result can be inaccurate due to unclear distinction between the ON-OFF states.

**Figure 3:**
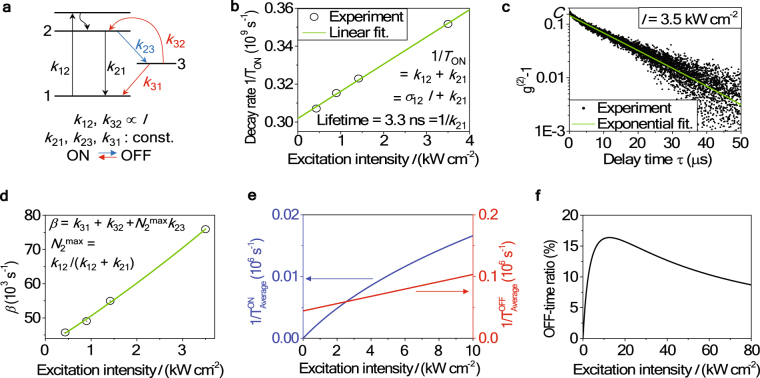
Blinking study by second-order correlation function *g*^(2)^(*τ*). (**a**) Energy band scheme of our target emitter. A meta-stable state of level 3 is considered for the blinking. (**b**) Decay rates 1/*T*_ON_ = *k*_12_ + *k*_21_ at four excitation pump intensities with a linear fitting line in green. *T*_ON_ is obtained by fitting the normalized g^(2)^(τ) in short-time scale (|*τ*| < 100 ns) shown in Fig. 1e. (**c**) The long-time g^(2)^(*τ* > 100 ns) is reconstructed from TTTR data obtained at an excitation intensity of 3.5 kW/cm^2^. (g^(2)^(*τ*) − 1) values as a function of the time delay *τ* is plotted in a semi-log scale and fitted with a single decaying exponential (green line) curve of ${g}^{2}(\tau )-1={\rm{C}}{e}^{-\beta \tau }$. The decay rates *β* at four excitation intensities are plotted in (**d**) with a function of $\beta \approx {k}_{31}+{k}_{32}\,+\,{N}_{2}^{max}{k}_{23}$. (**e**) The transition rates of ON → OFF (=$1/{T}_{Average}^{ON}={N}_{2}{k}_{23}$) and OFF → ON (=$1/{T}_{Average}^{OFF}={k}_{31}+{k}_{32}$) as a function of the excitation intensity. (**f**) The percentage ratio of the OFF time duration as a function of the excitation intensity under a continuous pumping.

To further look into the blinking behavior of the single emitter, we applied the second-order correlation function method that was reported by Fleury *et al*.^26^, in which they measured *g*^(2)^(*τ*) curves in short- and long-time ranges for different excitation intensities. To explain the anti-bunching and bunching of a single terrylene molecule at short- and long-times, respectively, they proposed a 3-level energy band scheme. This scheme included a meta-stable triplet state (level 3) associated with the intersystem crossing rate (*k*_23_) and the reverse intersystem crossing rate (*k*_32_ + *k*_31_)^26^. We adopted the same 3-level energy model for analyzing our target emitter because the ON/OFF probability distributions shown in Fig. 2e and f are well described by single exponentials^27^. This implies that there is *one* dominant energy level involved in the blinking processes, instead of the multi-levels as in many quantum dots which usually follow the power law in their blinking behaviors^9,10,24,25,27^. The schematic of adapted energy level structure is shown in Fig. 3a. We started our analysis by obtaining the transition rates *k*_12_ and *k*_21_, which were found from the short-time *g*^(2)^(*τ*) measurements. As shown in Fig. 1e, the short-time *g*^(2)^(*τ*) curves were fitted with double-side exponentials. The exponential decay constants for different excitation intensities were fitted to ${T}_{ON}^{-1}={k}_{12}+{k}_{21}$, where *k*_12_ is proportional to the pump intensity (*k*_12_ = *σ*_12_*I*) while *k*_21_ is a constant. Note that this fitting becomes valid when ${T}_{ON}^{-1}$ is much higher than the other decay rates of *k*_31_, *k*_32_, and *k*_23_. The validity of this assumption will be discussed after deriving all the values of decay constants. We found that ${k}_{12}=(0.14\pm 0.01)\times {10}^{8}\times I$ cm^2^/kW Hz, ${k}_{21}=(3.0\pm 0.1)\times {10}^{8}$ Hz, and the corresponding lifetime to be 3.3 ns (=1/*k*_21_). The other three decay rates of *k*_23_, *k*_31_, and *k*_32_ were smaller in their values compared to (*k*_12_ + *k*_21_), as confirmed later; they can be derived from the long-time *g*^(2)^(*τ* > 100 ns). *g*^(2)^(*τ*) over a long timescale was obtained from the time-tagged time-resolved (TTTR) data, as shown in Fig. 3c and was fitted with the function *g*^(2)^(*τ*) − 1 = C exp (−*βτ*), where C is a fitting constant and *β* is the decay rate associated with the metastable state (level 3). Under the assumption that *k*_12_ + *k*_21_ ≫ *k*_31_, *k*_32_, *k*_23_, we find $\beta \approx {k}_{31}+{k}_{32}+{N}_{2}^{max}{k}_{23}$, where ${N}_{2}^{max}={k}_{12}/({k}_{12}+{k}_{21})$. For the details, please refer to the Methods section. Here, *k*_31_ and *k*_23_ are constants and *k*_32_ = *σ*_32_*I*. Figure 3d displays *β* as a function of the excitation intensity. The fitting curve yielded the transition rates ${k}_{31}=(4.4\pm 0.1)\times {10}^{4}$ Hz, ${k}_{32}=(0.59\pm 0.02)\times {10}^{4}\times I$ cm^2^/kW Hz, and ${k}_{23}=(6.3\pm 1.7)\times {10}^{4}$ Hz. Acquired values of *k*_12_ and *k*_21_ were larger than those of *k*_23_, *k*_31_, and *k*_32_ by more than 3 orders of magnitude in our detection range, thus justifying our assumption. Using these transition rates, as shown in Fig. 3e, we calculated the average values of the ON/OFF time durations as ${T}_{Average}^{ON}=130$ μs and ${T}_{Average}^{OFF}=15$ μs at *I* = 3.5 kW/cm^2^. While ${T}_{Average}^{OFF}=15$ μs is close to 20 μs in Fig. 2f, but ${T}_{Average}^{ON}=130$ μs is much lower than the value obtained using PL intensity trajectory method, which was 1.26 ms. It is easy to understand that, in the PL intensity trajectory method, the average OFF state time duration is too short to be accurately detected resulting in a longer duration of the ON state. It is interesting, however, to note that the time bin size brings a larger error for the longer ${T}_{Average}^{ON}$ than for the shorter ${T}_{Average}^{OFF}$. Figure 3f shows the OFF-time ratio calculated utilizing the transition rates. At a specific pump intensity of 12 kW/cm^2^, the OFF-time ratio reaches its maximum value of 17%, reducing the emitted photon flux by the same amount due to the blinking. For more details about analyses and calculations, please refer to the Methods section.

**Figure 4:**
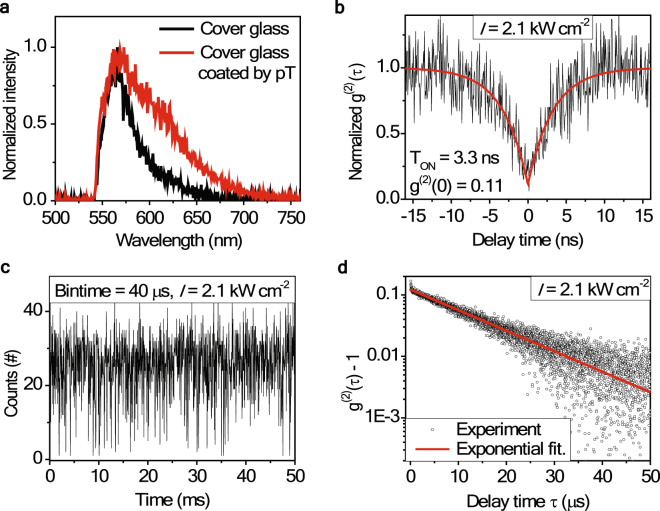
Silica defect center (DC) on cover glasses coated with a para-terphenyl crystal layer. (**a**) Spectral comparison of DCs from a bare cover glass (black curve) and from a cover glass coated with pT crystal (red curve). (**b**) Normalized *g*^(2)^(*τ*) of a DC from the cover glass coated with pT crystal at short time scale. It shows a good anti-bunching nature with *g*^(2)^(0) = 0.11. (**c**) The PL intensity trajectory of DC at excitation intensity *I* = 2.1 kW/cm^2^. (**d**) A semi-log plot of a long-time *g*^(2)^(*τ*) obtained at *I* = 2.1 kW/cm^2^. An exponential fitting is given in red.

In their recent work^22^, Rabouw *et al*. reported a non-blinking fluorescent defect centers (DCs) in a silica cover glass and gave an extensive discussion along with single emitters in previous studies. When we compared our single emitters with them, we detected several similarities. Both the reported data show multi-peaks, have similar peak positions, and the fluorescence lifetime is 3.3 ns in our case and 3.4 ns in their work. With the time bin size of 1 ms, we also recognized a non-blinking-like emission intensity trajectory; however, with an even smaller time bin size or by applying *g*^(2)^(τ) analysis we concluded that this emitter blinks with very short OFF durations (a few ~a few tens of μs). At this stage, we measured the fluorescent silica DC on cover glass. The DCs from cover glass gave much weaker fluorescence signal and bleached fast, not allowing us to measure enough photons for quantitative analysis. We could measure the PL spectrum (black curve in Fig. 4a) or short-time *g*^(2)^(*τ*) (inset of Fig. 1f) only at a few instances. In order to extend the surviving time of a single DC, we spin coated a para-Terphenyl (pT) crystal layer onto a clean cover glass. Though under this situation, it was not clear if the signal center originated from the cover glass or the pT layer, we could observe fluorescent signal with a similar peak position but broader emission band, as shown in Fig. 4a. If we accept this emitter as the DC on the cover glass, the broadened band possibly can be explained by the additional phonon broadening by the pT crystal layer. The short-time *g*^(2)^(*τ*) of this DC showed a good anti-bunching with *g*^(2)^(0) = 0.11 and the fluorescence lifetime of 3.3 ns. Figure 4c displays the PL intensity trajectory at an excitation intensity *I* = 2.1 kW/cm^2^ with a time bin size of 40 μs. Although the fluctuation between ON-OFF state is not clear enough, the long-time *g*^(2)^(*τ*) shown in Fig. 4d clearly demonstrates a bunching behavior suggesting that this DC also has a fast blinking behavior. However, when we applied the same 3-level model we found a larger error than for the impurity in toluene. More studies for improving the photon detection capability and for finding a more suitable energy band scheme are underway.

## Conclusion

In this study, we have reported a bright single fluorescent emitter contained in toluene. Its emission spectrum showed a progression of peaks separated by ~167 meV, with the main peak position varying from 2.03 eV (610 nm) to 2.13 eV (580 nm). The fluorescence lifetime was found to be around 3.3 ns, and emitted photons were found to show a strong anti-bunching. Blinking studies by two methods, namely, the PL intensity trajectory and the *g*^(2)^(*τ*) analysis, revealed that this emitter blinked very fast with an average “OFF” time duration of only a few tens of μs. A 3-level energy band scheme was shown to plausibly explain our measurements. The blinking parameters were derived in order to discuss the details of its blinking behavior.

We compared the photon emission properties of the impurity in toluene with other reported single fluorescent centers including the DC on the silica cover glass. Our results confirm that this impurity in toluene is clearly distinguishable from silica DCs. Further, its blinking was too fast to be properly studied by the conventional PL intensity trajectory method. Toluene is a very common solvent for the solutions of colloidal QDs and single molecules; therefore, researchers should be very careful not to confuse background emitters like this impurity in toluene with their target emitters.

## Methods

### Sample preparation

Microscope cover glasses (Marienfeld, borosilicate glass, 18 × 18 mm^2^) were immersed in an Aqua Regia (HCl/HNO_3_ 3:1 in volume ratio) for 30 mins for cleaning, followed by 5-min sonication in DI water, and finally dried with N_2_.

Toluene solutions were purchased from two manufacturers; Sigma-Aldrich (ACS reagent, ≥99.5%) and Daejung (>99.5%). Few drops of toluene on the cover glasses were evaporated on a hot plate at 86 °C. Para-terphenyl (Sigma-Aldrich, >99.5%) was dissolved in toluene (Sigma-Aldrich) with a concentration of 1.2 mg/ml and then spin coated on cleaned cover glasses at 2000 rpm for 5 s.

### Sample measurement

In order to excite a single emitter from the sample, we used a cw 532 nm laser focused by an oil objective lens (Nikon Plan Apo VC, 100×, NA = 1.40). Three other excitation sources of 405 nm, 448 nm, and 594 nm lasers were used only in measuring the absorption cross section. The stream of emitted photons was collected by the same objective lens, separated from the excitation light via a long-pass filter (Semrock, 532 nm), and guided to a CCD camera or Hanbury Brown-Twiss (HBT) set-up where photons were detected by the two single photon counting avalanche photo diodes (Perkin Elmer, SPCM-AQ4C). Spatial filtering was applied to select the signal only from single emitter position in order to reduce the background. The short-time g^(2)^(|τ| < 100 ns) was measured by the start-stop mode of Pico-Harp (model300, PicoQuant, 4 ps time resolution) and the long-time *g*^(2)^(100 ns < τ < 50 µs) was reconstructed from the intensity time trace recorded by TTTR mode of Pico-Harp. Each TTTR dataset was measured for 10 s. All the measurements were performed at room temperature.

### PL intensity trajectory analysis by threshold method

The PL intensity trajectory was analyzed by a threshold method. In brief, the threshold intensity was selected (red dashed in Fig. 2b and c) and the intensities above(below) the threshold were considered as “ON” (“OFF”). To calculate the probability distributions P(*τ*_ON/OFF_) for duration times of the ON/OFF periods (*τ*_ON/OFF_) we divided the number of occurrences of a given event by the average time duration between the neighboring pre- and post-events^25^, $P({\tau }_{ON/OFF})=\frac{N({\tau }_{ON/OFF})}{{N}_{ON/OFF}^{tot}}\frac{1}{\delta {\tau }_{ON/OFF}}$.

### Transition rate equations, *g*^(2)^(τ), and blinking analysis

The rate equations of the 3-level model adapted in this work can be written as:

1 $$\frac{d}{dt}(\begin{array}{c}{N}_{1}\\ {N}_{2}\\ {N}_{3}\end{array})=(\begin{array}{ccc}-{k}_{12} & {k}_{21} & {k}_{31}\\ {k}_{12} & -{k}_{21}-{k}_{23} & {k}_{32}\\ 0 & {k}_{23} & -{k}_{31}-{k}_{32}\end{array})(\begin{array}{c}{N}_{1}\\ {N}_{2}\\ {N}_{3}\end{array})$$

Here, *k*_12_ and *k*_32_ are proportional to the pump intensity while the other three rates are constants. *N*_*i*_ (*i* = 1, 2, 3) denotes the population density at *i*-level with a constraint of $\sum {N}_{i}=1$. *g*^(2)^(τ) is proportional to *N*_2_(*τ*) with the condition *N*_1_(0) = 1. By the normalization condition of *g*^(2)^(τ → ∞) = 1, *N*_2_(τ)/*N*_2_(∞) = *g*^(2)^(τ). By measuring and fitting short-time *g*^(2)^(*τ*) for different pump intensities, we arrived at $\frac{1}{{T}_{ON}}={k}_{12}+{k}_{21}={\sigma }_{12}I+{k}_{21}$, which depends linearly on excitation intensity *I* with *k*_21_ being the *y*-intercept. Here, *T*_ON_ is the decay constant of exponential fitting shown in Fig. 1e. By solving Eq. (1), we found that the decay rate of the long-time *g*^(2)^(*τ* > 100 ns) could be described as follows: ${g}^{2}(\tau )-1\approx {\rm{C}}{e}^{-\beta \tau }$, where

2 $$\beta \approx {k}_{31}+{k}_{32}+{N}_{2}^{max}{k}_{23}={k}_{31}+{\sigma }_{32}I+\frac{{k}_{12}}{{k}_{12}+{k}_{21}}{k}_{23},$$

or

3 $$\beta \approx ({k}_{31}+{\sigma }_{32}I)\times (1+\frac{\alpha }{1+\frac{{k}_{31}}{{\sigma }_{12}I}}),$$

where $\alpha =\frac{-\,C}{-\,1+A+AC}$. To derive Eq. (3) from Eq. (2), we use the condition of that short- and long-time *g*^(2)^ curves should be continuous to each other. Values of *A* and *C* were determined from short- and long-time *g*^(2)^ curves, as shown in Figs 1e and 3c, respectively. By fitting *β* values obtained for different pump intensities with Eq. (3) we derived *k*_31_ and *σ*_32_. The value of *k*_23_ is finally derived from eq. (2) by inserting pre-determined values of *k*_12_, *k*_21_, *σ*_32_, and *k*_31_.

The average duration times of the ON (OFF) states were calculated as follows:

4 $${T}_{Average}^{ON}=\frac{1}{{N}_{2}{k}_{23}}$$

and

5 $${T}_{Average}^{OFF}=\frac{1}{{k}_{31}+{\sigma }_{32}I}.$$

The OFF-time ratio, shown in Fig. 3f, was obtained as *N*_3_(*τ* → ∞). For this duration of time, the emitter stays in its OFF-state and does not emit photons. Note that this OFF-time ratio is valid only for the cw-mode excitation case and not for the pulsed excitation cases.
